# Identification of the prognostic value of LACTB2 and its correlation with immune infiltrates in ovarian cancer by integrated bioinformatics analyses

**DOI:** 10.1186/s40001-024-01762-2

**Published:** 2024-03-12

**Authors:** Weiwei Cao, Chao Wang, Yue Zhang, Jiani Yang, Xiaomei Luo, Yaqian Zhao, Meixuan Wu, Shanshan Cheng, Yu Wang

**Affiliations:** 1 Department of Gynecology, Shanghai First Maternity and Infant Hospital, School of Medicine, Tongji University, Shanghai, 200092 China; 2 Shanghai Key Laboratory of Maternal Fetal Medicine, Shanghai Institute of Maternal-Fetal Medicine and Gynecologic Oncology, Shanghai First Maternity and Infant Hospital, School of Medicine, Tongji University, Shanghai, 200092 China; 3 Department of Obstetrics and Gynecology, Ren Ji Hospital, Shanghai Jiao Tong University School of Medicine, Shanghai, 201204 China

**Keywords:** LACTB2, Ovarian cancer, Immunology, Prognosis

## Abstract

Ovarian cancer (OC) is one of the most common reproductive tumors in women, whereas current treatment options are limited. β-lactamase-like-protein 2 (LACTB2) has been observed to be associated with various cancers, but its function in OC is unknown. Therefore, we evaluate the prognostic value and the underlying function of LACTB2 in OC. In this study, high expression of LACTB2 was observed in OC compared with normal controls. Kaplan–Meier Plotter analysis revealed that overexpressed LACTB2 is strongly correlated with poor prognosis. We conducted GO/KEGG analysis to investigate the potential biological function of LACTB2 in OC. GESA analysis showed that LACTB2 was closely related to immune-related pathways. Subsequently, we explored the relationship between LACTB2 and 24 types of immune cells in OC. The results suggested that LACTB2 was positively associated with multiple tumor-infiltrating immune cells. Importantly, LACTB2 may modulate immune cell infiltration in OC to influence prognosis. In conclusion, LACTB2 can be used as a promising prognostic biomarker and immunotherapy target for OC.

**Supplementary Information:**

The online version contains supplementary material available at 10.1186/s40001-024-01762-2.

## Introduction

Ovarian cancer (OC) is a common lethal female reproductive disease which represents a leading cause of deaths in women worldwide, with over 200,000 deaths reported per year [1]. According to recent studies, early intervention of OC can achieve a 5-year survival rate of 70–80%. However, the prognosis for advanced patients deteriorates significantly, with the 5-year survival rate less than 30% [2, 3]. OC in early stage has no specific symptoms and few screening biomarkers are available in clinical practice, thus OC is difficult to be diagnosed in time. Recent advances in high-throughput sequencing and transcriptomic studies allow the identification of numerous critical driver genes. Therefore, there is an urgent need to determine a reliable biomarker to facilitate early diagnosis, prognosis prediction, and serve as potential therapeutic target for OC.

β-lactamase-like-protein 2 (LACTB2), a human mitochondrial endoribonuclease, is a member of glyoxalase II family which is responsible for the regulation of mitochondrial function and cell viability [4]. Several researches have confirmed that mitochondrial transcripts can disrupt cellular metabolism and thus promote tumor progression [5, 6]. Levy et al. reported that alteration of LACTB2 protein expression destroys mitochondrial function, and then causes morphological changes and cell death [7]. Overexpression of LACTB2 is observed in nasopharyngeal carcinoma and is associated with radioresistance which leads to unfavorable prognosis [8]. Another study revealed that genetic variation of LACTB2 contributes to colorectal cancer formation [9]. However, there is a lack of research on the correlation between LACTB2 expression and OC.

In our study, we investigated the expression of LACTB2 in OC and its potential biological function. RNA-seq data of LACTB2 in OC were downloaded from TCGA and GEO datasets to compare the LACTB2 expression level in normal ovarian tissue and ovarian tumor tissue. We also downloaded immunohistochemical images from HPA to investigate the protein expression of LACTB2 in OC. Next, we demonstrated the feasibility of LACTB2 as a reliable biomarker to predict the prognosis of OC patients. We found that LACTB2 expression was upregulated in OC, which contributes to poor prognosis of OC patients. In addition, we identified the differentially expressed genes (DEGs) by comparing gene profiles in low- and high-LACTB2 expression groups. Subsequently, we conducted enrichment analysis based on the DEGs to predict the biological functions of LACTB2. The results suggested that LACTB2 may be involved in immune processes. Immune cell infiltration plays an important role in the overall survival of OC patients [10, 11]. Thus, we further investigated the correlation between LACTB2 and immune cells infiltration in OC and found that Th2 cells infiltration was significantly high in samples with high LACTB2 expression. Our study reported the potential role of LACTB2 in tumor immunology, thereby highlighting the underlying mechanism by which LACTB2 causes poor prognosis in OC.

## Materials and methods

### Gene expression analysis

We downloaded RNA-sequencing data of LACTB2 for 33 cancer types from the Cancer Genome Atlas (TCGA) web portal (http://cancergenome.nih.gov/). The full name of 33 cancers and their corresponding abbreviations are summarized in Additional file 1: Table S1. We then analyzed LACTB2 expression in pan-cancer level using Gene Expression Profiling Interactive Analysis (GEPIA, http://gepia.cancer-pku.cn/), which can visualize the relative expression of the gene of interest in normal and tumor samples. Users can customize statistical methods and thresholds to analyze the different expression of genes in specific database [12]. Here, normal/tumor differential expression of LACTB2 in OC was also investigated by GEPIA. We calculated log2 transcript per million (TPM) value of LACTB2. The results were presented by the log2(TMP + 1) scale. Moreover, we explored LACTB2 expression in OC based on Gene Expression Omnibus (GEO) database (https://www.ncbi.nlm.nih.gov/geo/), including GSE12470, GSE18250, GSE137238, and GSE51088. UALCAN is a comprehensive online-portal (http://ualcan.path.uab.edu), which allows comparison of the differences in specific genes expression in normal and tumor tissue, as well as in multiple cancer subgroups based on various clinicopathological features. Users can search for genes of interest in selected cancer types to analyse the expression and survival information of the queried genes. UALCAN can link to the results of gene expression analysis and survival analysis. We assessed the relationship between LACTB2 gene expression and clinical characteristics, including cancer stage, tumor grade, ethnicity, age, etc.[13].

### Protein expression analysis

The Human Protein Atlas (HPA, http://www.proteinatlas.org/) examines the expression of 26,000 human proteins using immunoassay techniques [14]. In this study, we obtained immunohistochemical images of LACTB2 from HPA to observe their distribution and protein expression level in normal and OC tumor tissue. Additionally, proteomic data of LACTB2 were acquired from Clinical Proteomic Tumor Analysis Consortium (CPTAC) and then evaluated by UALCAN.

### Identification of differentially expressed genes (DEGs)

We analyzed LACTB2 expression values from TCGA database to screen significant DEGs in OC. The datasets were categorized as low- and high-LACTB2 expression groups using a median threshold. We used the R package edgeR-based DESeq2 (version 3.10) software to perform an unpaired Student's t-test to compare the differences in expression profiles between the two groups [15]. Genes with an absolute fold change log (FC) > 1.5 and *P*_adj_ < 0.05 would be counted with statistically significant DEGs. The results were visualized as volcano plots. The top 50 genes that were positively and negatively associated with LACTB2 were clustered in the heat map drawn by the R Statistics 3.6.3 software.

### Functional enrichment analysis

Functional enrichment analysis of LACTB2 in OC was conducted using TCGA database to visualize the interactions of coexpressed genes and predict the role of LACTB2. We performed Gene Ontology (GO) and Kyoto Encyclopedia of Genes and Genomes (KEGG) analysis using the R package “clusterProfiler”. Gene Set Enrichment Analysis (GSEA) is a computational tool which can enrich the genomes that share biological functions, chromosomal locations or regulation, thus unravelling important biological processes in cancer [16]. For this purpose, the enrichment score of DEGs was calculated and then analyzed their statistical significance. Finally, the significance level was adjusted based on multiple hypothesis test. In this study, we performed GESA analysis of LACTB2 expression value using the gseGO, gseKEGG, and gsePathway functions of the R software. Eventually, protein network of LACTB2 was visualized by STRING analysis (https://cn.string-db.org/).

### Survival prognosis analysis

The Kaplan–Meier Plotter database (https://kmplot.com/analysis/) was used to evaluate the prognostic value of LACTB2 [17]. The expression data extracted from TCGA were divided into high and low LACTB2 expression groups based on best-cutoff value calculated by algorithm. The overall survival (OS), progression-free survival (PFS), recurrence-free survival (RFS) was determined in different LACTB2 expression groups. We calculated HR with 95% Cl and *P*-value, which were shown in the Kaplan–Meier Plotter. In addition, we further evaluated disease-free survival (DFS) of LACTB2-altered and unaltered groups to confirm the impact of genetic alteration on OC patient’s prognosis. Eventually, we obtained OC microarray data from GEO database and predicted the relationship of LACTB2 expression with OS using the PrognoScan database (http://www.abren.net/PrognoScan/), which is a publicly available platform of various cancer microarray datasets [18]. PrognoScan is conducted using minimum *P*-value approach to evaluate the correlation between gene expression and prognosis.

### Immune cell infiltration analysis

The relationship between tumor-infiltrating immune cells and LACTB2 expression in OC was identified by the single sample Gene Set Enrichment Analysis (ssGSEA) from the GSVA R package and Tumor IMmune Estimation Resource (TIMER; http://timer.comp-genomics.org/) [19, 20]. TIMER is developed by the integration of multiple state-of-the-art algorithms. It allows exploring the expression profiles of tumors in TCGA and predicting various correlations between immune infiltration and genetic traits. In this study, 24 types of immunocytes were included and we quantified the relative level of infiltration based on the gene expression profile [21]. Spearman correlation tests were conducted to investigate the relationship between LACTB2 expression in OC and the abundance of immunocytes infiltration levels. Wilcoxon rank-sum tests were conducted to confirm the differential level of immune cell infiltration in high- and low-LACTB2 expression groups. Additionally, we evaluated multiple immune cells infiltration level using TIMER, and then investigated the correlation of LACTB2 expression relying on CIBERSORT and XCELL algorithms.

### cBioPortal analysis

The cBioPortal for Cancer Genomics (http://cbioportal.org) is a reliable online resource enabling researchers to analyze genetic variations across multiple cancer genomic data [22]. It provides an integrative platform of multiple data types at the gene level, which allow users to query specific biological events, such as mutation, deletion, and amplification, in custom samples. In this literature, we used the cBioPortal to assess genetic alterations in LACTB2, including amplifications, deep deletions, and mutations, across 32 cancer types from TCGA. An overview of LACTB2 alterations for each OC patient was generated by OncoPrint.

### Predictive value analysis

To investigate the feasibility of LACTB2 as a diagnostic biomarker candidate, we drew Receiver operating curve (ROC) plotter using R package (version 3.6.3). The area under ROC was calculated to evaluate the predictive value of LACTB2 in distinguish OC patients from healthy women.

## Results

### Analysis of LACTB2 expression in pan-cancer

**Fig. 1:**
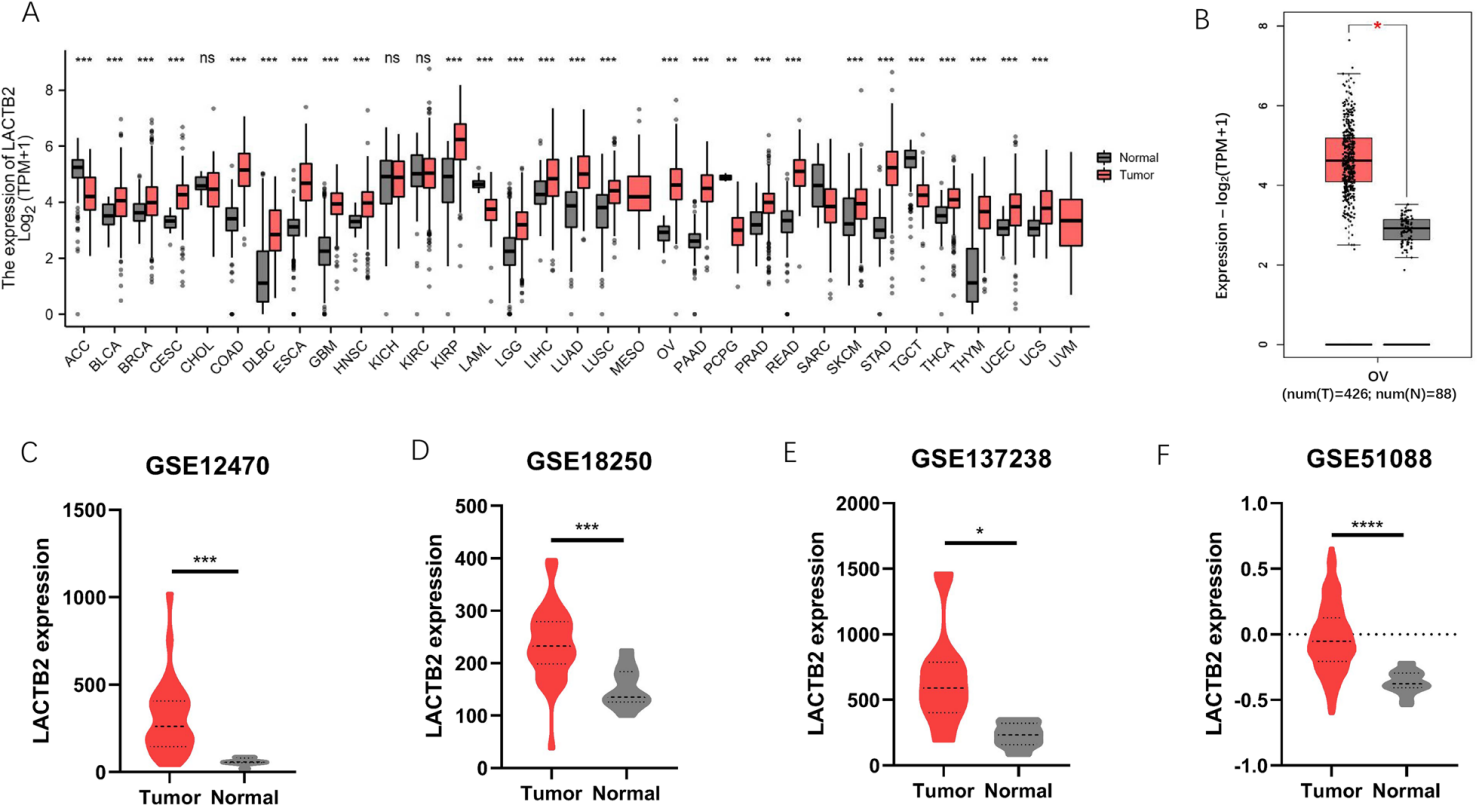
Pan-cancer analysis of LACTB2 expression. **A** LACTB2 expression in 33 types of cancer and normal tissue from TCGA. **B** LACTB2 expression in OC and normal tissue. **C–F** LACTB2 expression in OC and normal tissue from GSE12470, GSE18250, GSE137238, and GSE51088, respectively

We obtained data from TCGA and analyzed the mRNA expression of LACTB2 in 33 types of cancer using GEPIA. Figure 1A shows that LACTB2 was highly expressed in most cancers, especially in BLCA, BRCA, CESC, CHOL, COAD, DLBC, ESCA, GBM, HNSC, KIRP, LGG, LIHC, LUAD, LUSC, OC, PAAD, PRAD, READ, SKCM, STAD, THCA, THYM, UCEC, UCS. LACTB2 expression in OC was particularly confirmed using 426 OC patients and 88 normal women. The result reported the overexpression of LACTB2 in OC (*P* < 0.05) (Fig. 1B). Additionally, the expression of LACTB2 in OC was assessed in the GEO database, including GSE12470, GSE18250, GSE137238, and GSE51088, and the results showed that the expression level was significantly higher in tumor tissue than in normal tissue (Fig. 1C–F). To further validate the diagnostic value of LACTB2, we performed the receiver operating curve (ROC) analysis, and the area under the ROC curve (AUC) was 0.981 (Additional file 1: Fig. S1). This result may indicate that LACTB2 can be used as a diagnostic biomarker to distinguish OC patients from healthy women.

### Analysis of LACTB2 in OC patients

**Fig. 2:**
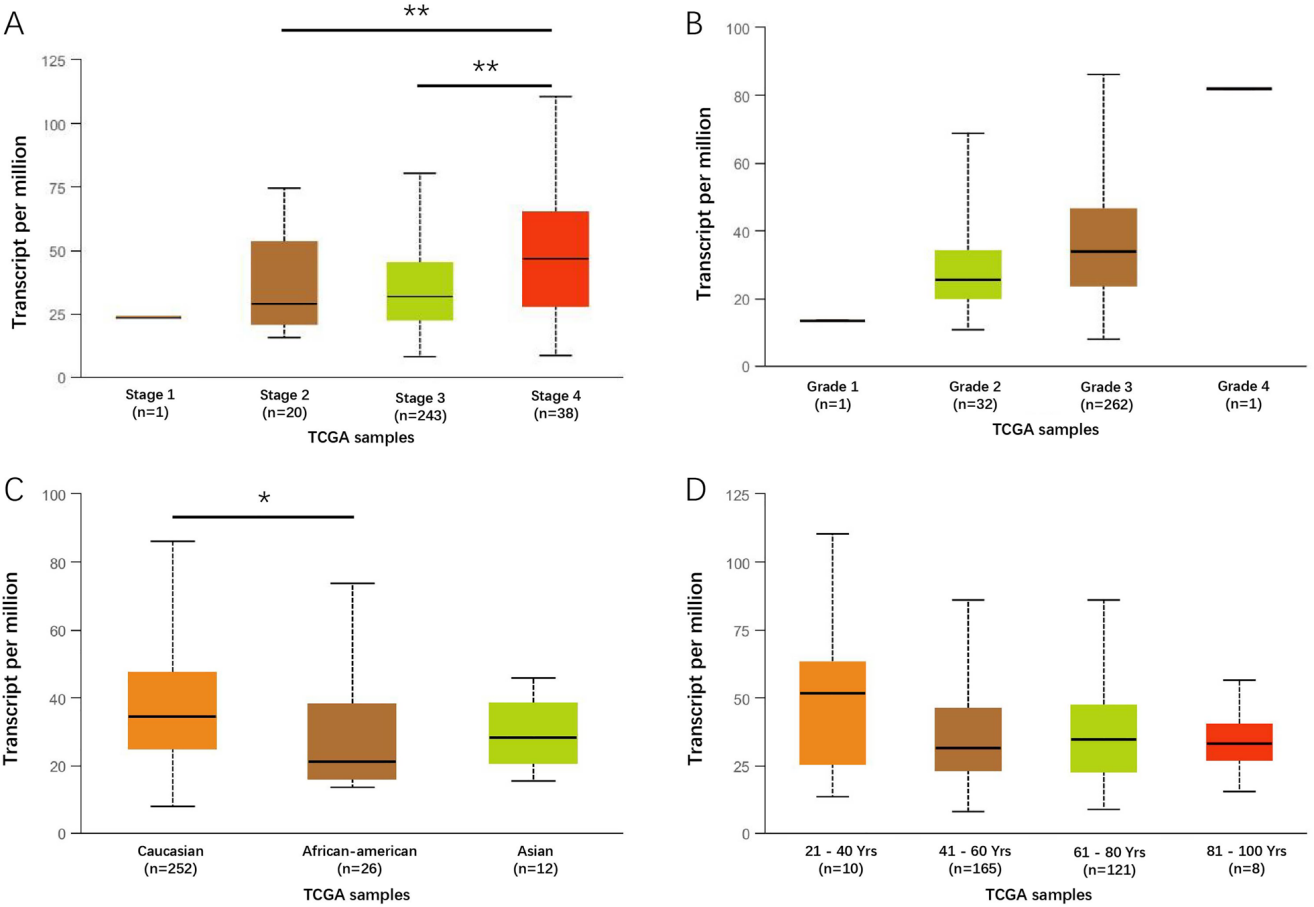
LACTB2 transcription level in OC. **A** Expression of LACTB2 in terms of cancer stage. **B** Expression of LACTB2 in terms of tumor grade. **C** Expression of LACTB2 in terms of patients’ race. **D** Expression of LACTB2 in terms of patients’ age

We used UALCAN database to investigate the relationship between LACTB2 expression and specific clinical features in patients with OC, including FIGO stage, pathologic grade, race, and patient age. The result demonstrated that LACTB2 expression was higher in FIGO IV patients compared to FIGO II and FIGO III (Fig. 2A). Patients with high pathological grading had higher LACTB2 expression than those with low pathological grading (Fig. 2B). Moreover, we found that LACTB2 expressed highly in Caucasian patients when compared with African-american and Asia (Fig. 2C). However, Fig. 2D reported no correlation of LACTB2 with the age of patients.

### Protein expression of LACTB2 in OC patients

**Fig. 3:**
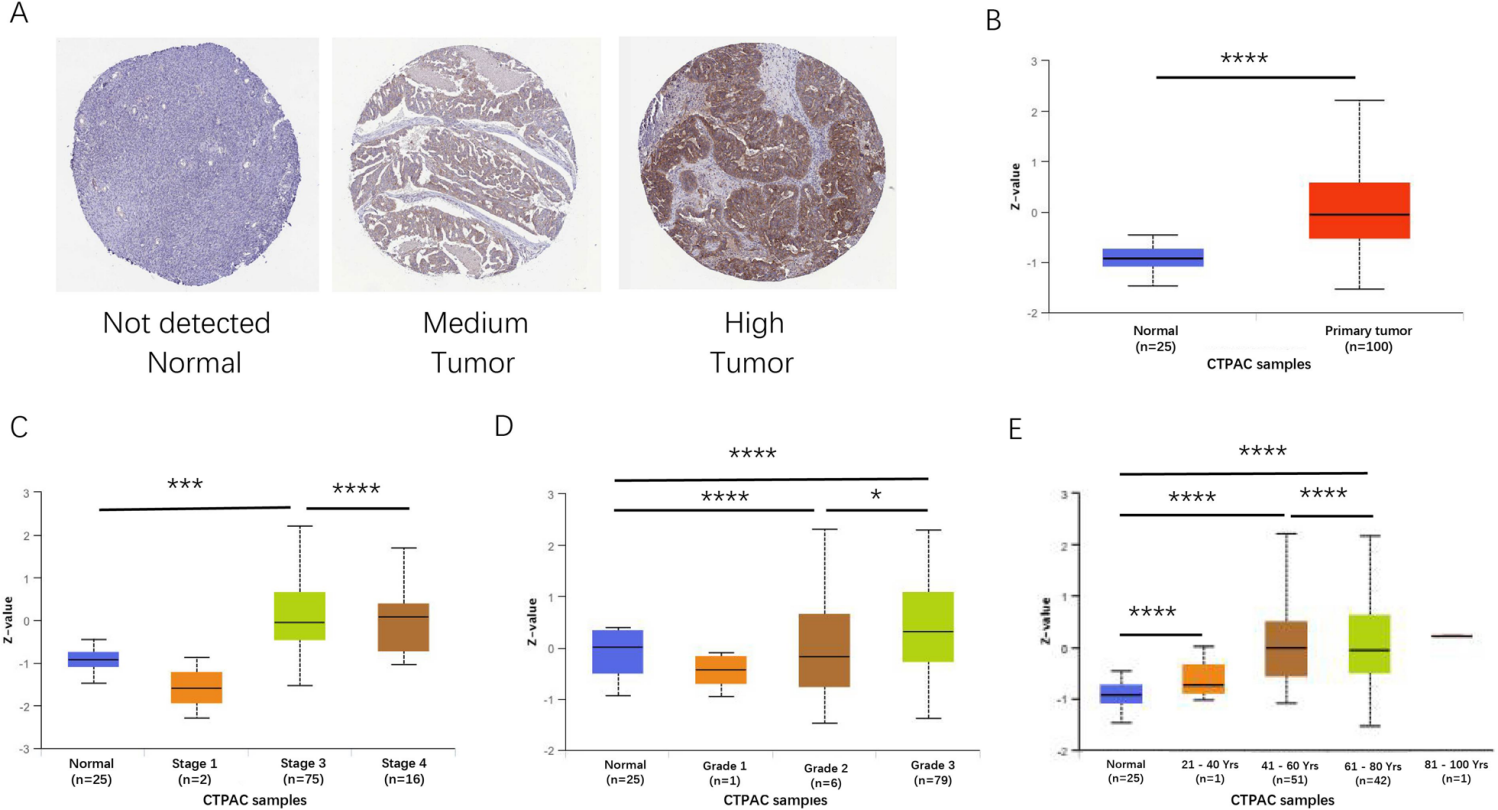
Protein expression of LACTB2 in OC. **A** Representative immunohistochemical images of LACTB2 expression in OC tissue and normal tissue. **B** Comparison of protein levels of LACTB2 in OC sample and normal sample. **C** Protein levels of LACTB2 based on cancer stage. **D** Protein levels of LACTB2 based on tumor grade. **E** Protein levels of LACTB2 based on patients’ age

We obtained the images of immunohistochemical staining sample in OC patients and normal women from HPA database. The expression of LACTB2 can be seen in OC tissue while it was not detected in normal tissues (Fig. 3A). CTPAC database confirmed protein levels of LACTB2 were notably high in primary tumor samples than in normal tissues (Fig. 3B). We further analyzed LACTB2 expressions based on cancer stage, tumor grade, and patients age, respectively. Stage IV patients showed higher LACTB2 expression than stage III (Fig. 3C). LACTB2 protein expression was higher in Grade 3 OC than in Grade 2 OC (Fig. 3D). In CPTAC database, the expression of LACTB2 increased in older patients (Fig. 3E).

### Prognostic value of LACTB2 expression

**Fig. 4:**
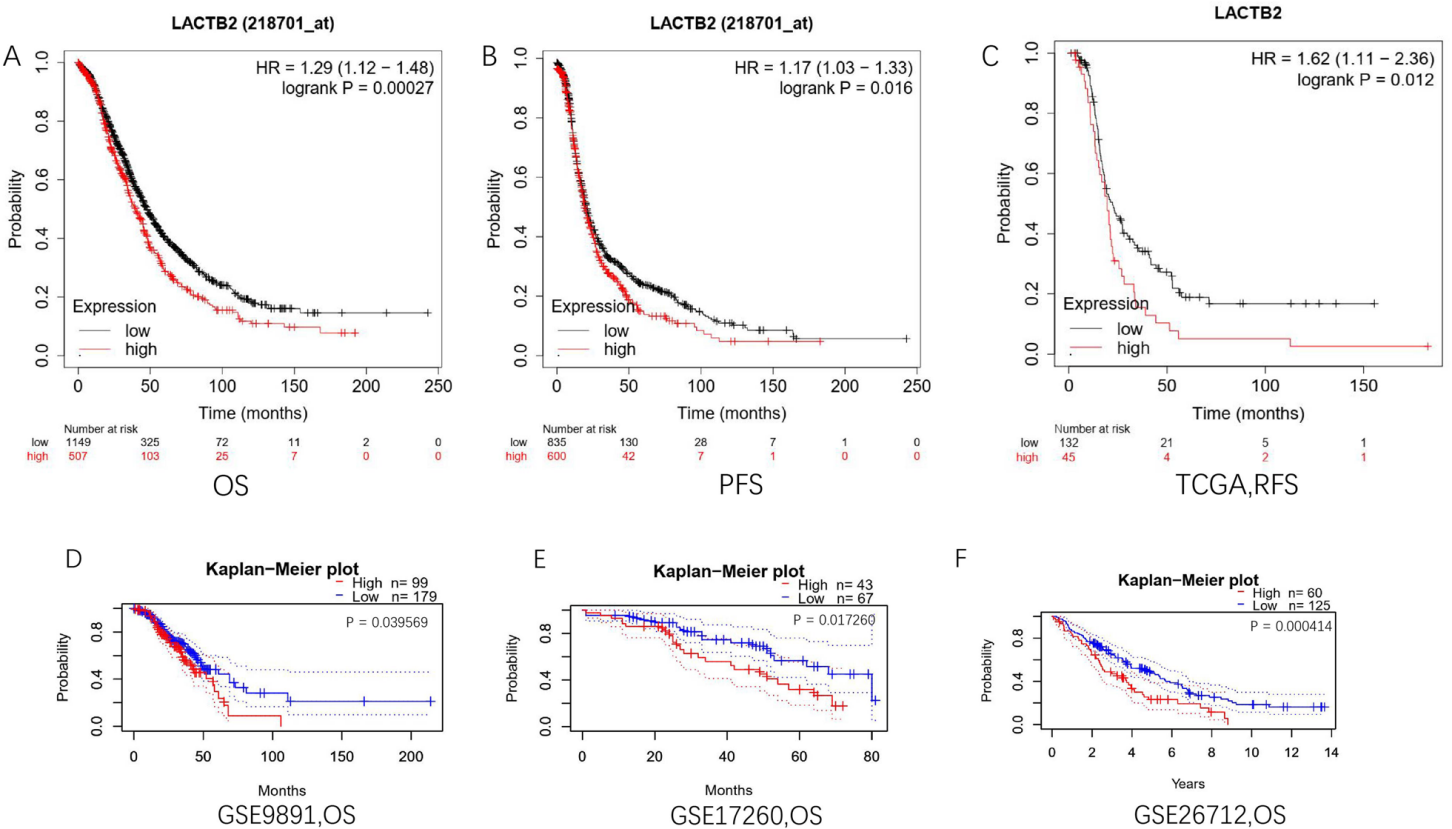
Prognostic value of LACTB2 expression in OC. **A** OS in OC patients from TCGA. **B** PFS in OC patients from TCGA. **C** RFS in OC patients from TCGA. **D** OS in OC cohort GSE9891. **E** OS in OC cohort GSE17260. **F** OS in OC cohort GSE26712

To demonstrate the clinical significance of elevated LACTB2 expression in OC, we performed survival analysis using multiple independent patient cohorts. Firstly, 1656 patients with overall survival (OS) data and 1435 patients with progression-free survival (PFS) data were included and analyzed via Kaplan–Meier plotter in Fig. 4A and B. The results reported that the patients with high LACTB2 expression had shorter OS and PFS. In TCGA database, high-LACTB2 expression group showed a decreased RFS [HR = 1.62(1.11–2.36), *P* = 0.012] compared to the low-LACTB2 group (Fig. 4C). These findings were further verified in 3 different GEO databases (GSE9891, GSE17260, GSE26712). All datasets indicated that elevated LACTB2 expression was correlated with poor OS in OC patients (Fig. 4D–F). The prognostic value of LACTB2 was additionally verified in various cancers, including BRCA, HNSC, PAAD, ESCA, and UCEC. BRCA patients with high LACTB2 expression showed poor OS and RFS (Additional file 1: Fig. S3A, B). Similar results were reported in PAAD and HNSC patients (Additional file 1: Fig. S3C–F). High expression of LACTB2 was notably related to worse OS in ESCA and UCEC patients (Additional file 1: Fig. S3G, H). We can conclude that LACTB2 can be used as a prognostic biomarker.

### Enrichment analysis of LACTB2

**Fig. 5:**
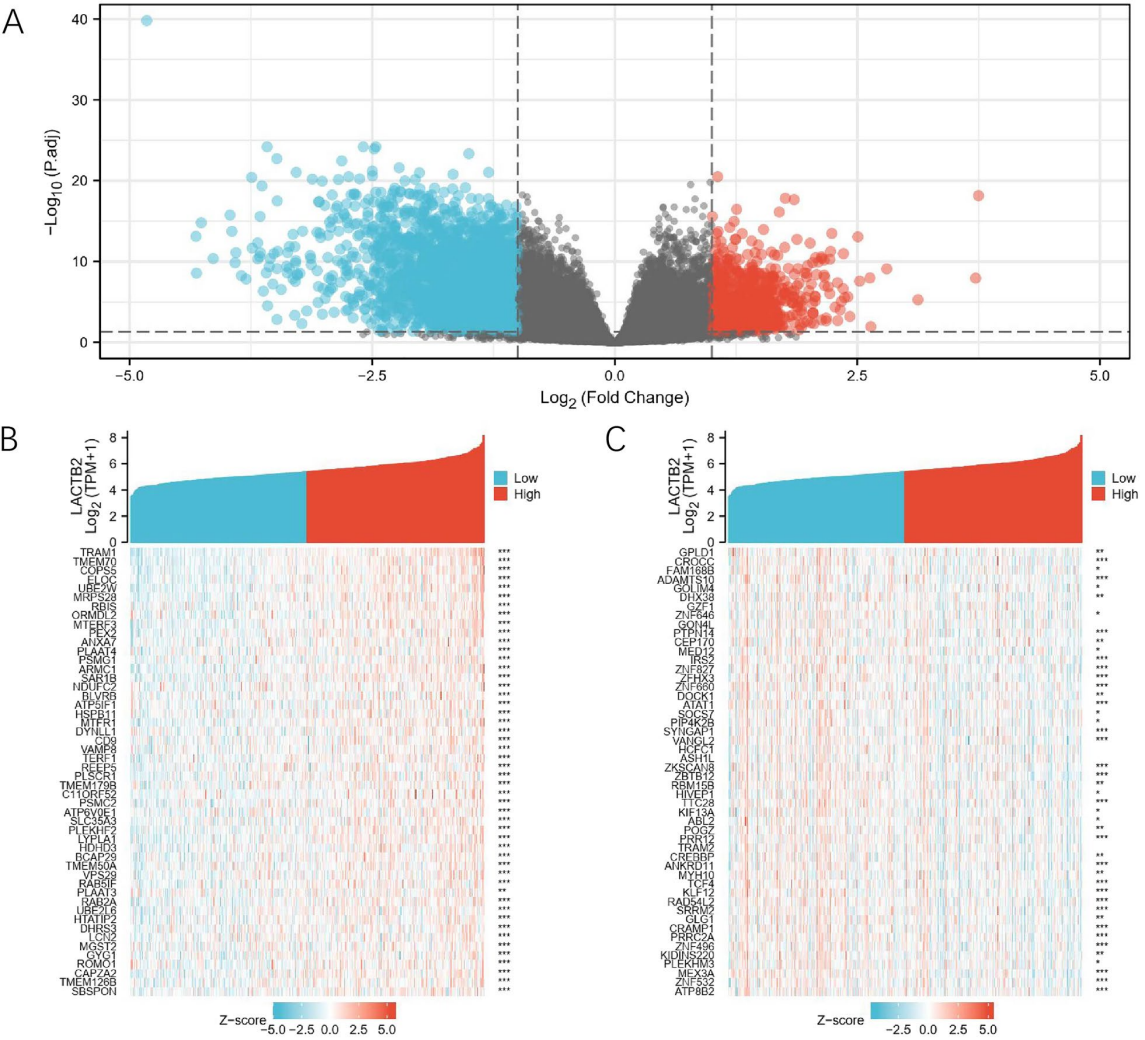
Identification of LACTB2-related DEGs. **A** Volcano plot of the DEGs between LACTB2 high and low groups. **B** Top 50 genes positively correlated with LACTB2 expression. **C** Top 50 genes negatively correlated with LACTB2 expression

As the above findings suggested that LACTB2 expression was negatively related to OC survival, we tried to determine the signaling pathways involved in LACTB2 and thus investigate the specific role of LACTB2 in OC development. We first obtained LACTB2 expression data from TCGA database and divided them into LACTB2 high and low groups. The gene expression profiles of LACTB2 in two groups were compared and the volcano plot reported all differentially expressed genes (DEGs) (Fig. 5A). Furthermore, we performed hierarchical clustering analysis. The top 50 DEGs positively correlated with LACTB2 expression are shown in Fig. 5B, while those negatively correlated are in Fig. 5C. The results demonstrated that high and low LACTB2 expression groups had significantly different DEG patterns.

**Fig. 6:**
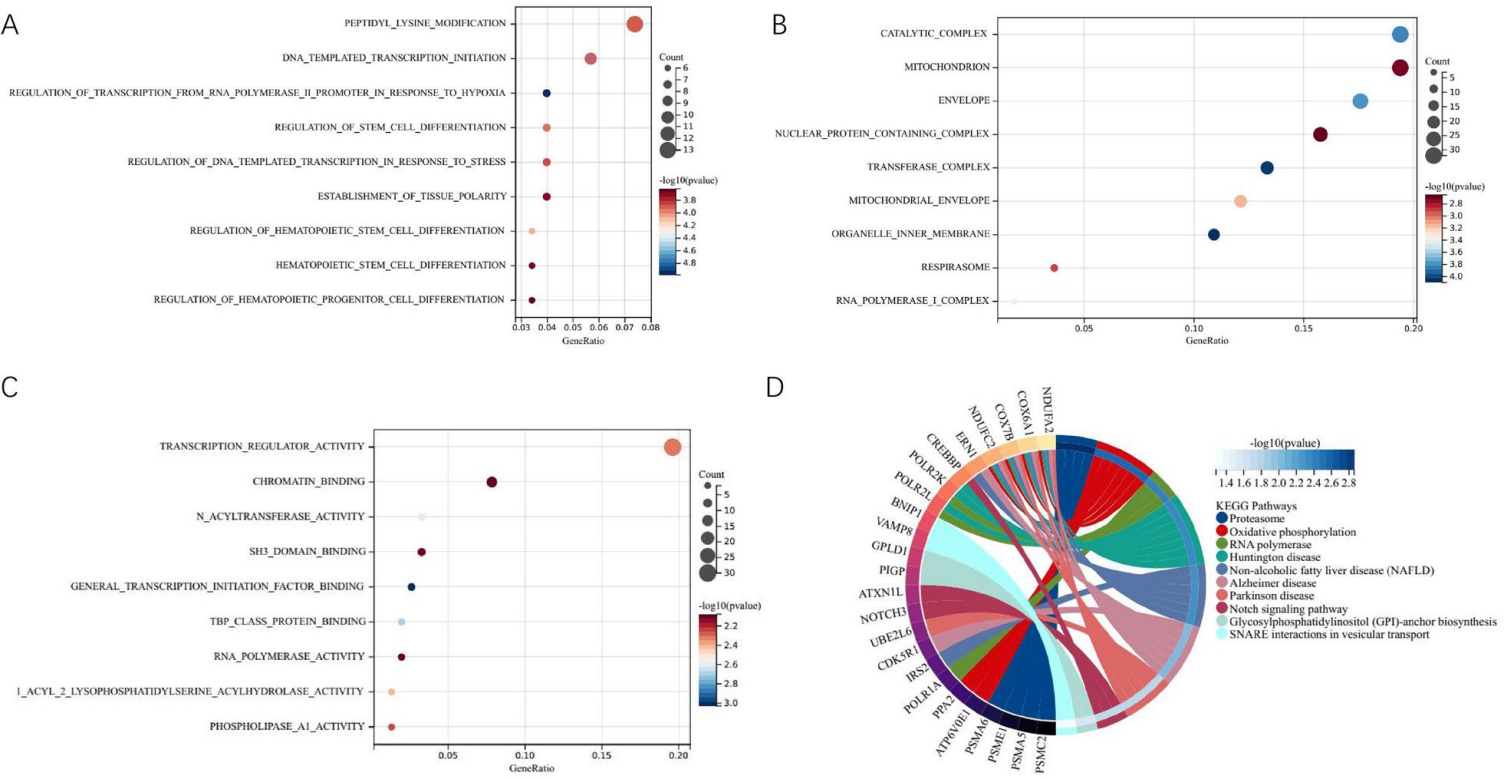
GO/KEGG analysis of genes associated with LACTB2. **A–C** Enriched GO terms in the BP, CC, and MF. **D** KEGG analysis of genes associated with LACTB2

**Fig. 7:**
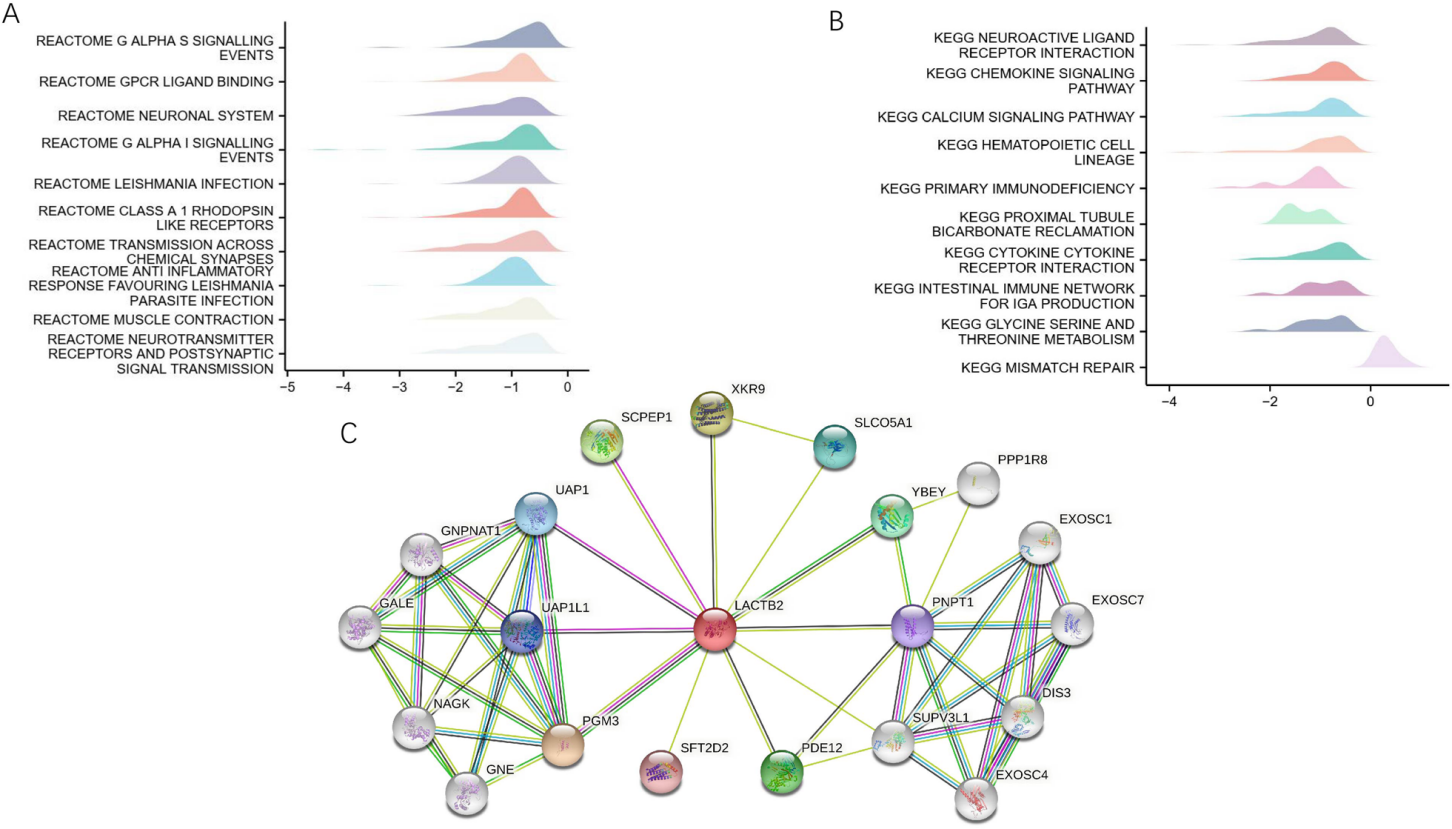
Pathway enrichment analysis of LACTB2 in OC. **A**, **B** GESA results of pathways associated with LACTB2, including Reactome pathway (**A**) and KEGG pathway (**B**). **C** Protein interactions of LACTB2

We then utilized GO and KEGG pathway enrichment analysis of DEGs to further predict the relationship between typical DEGs and LACTB2. GO analysis revealed the DEGs that remarkably enriched in biological process (BP), cellular component (CC), molecular function (MF). The top 9 significant pathways of BP, CC, MF are presented in Fig. 6A–C. KEGG pathway analysis showed a complex interaction network among LACTB2 coexpressed genes. LACTB2 was closely correlated with proteasomes, including PSME1, PSMA6, PSMA5, PSMC2 (Fig. 6D). In addition, we used Gene Set Enrichment Analysis (GSEA) to enrich the KEGG and Reactome pathways associated with LACTB2 in OC. Reactome pathway analysis reported top 10 enriched pathways, including G ALPHA S signaling events, GPCR ligand binding, neuronal system, G ALPHA I signaling events, leishmania infection, class A 1rhodopsin like receptors, transmission across chemical synapse, anti-inflammatory response favouring leishmania parasite infection, muscle contraction, and neurotransmitter receptors and postsynaptic signal transmission (Fig. 7A). Meanwhile, KEGG pathway was enriched in neuroactive ligand receptor interaction, chemokine signaling pathway, calcium signaling pathway, hematopoietic cell lineage, primary immunodeficiency, proximal tubule bicarbonate reclamation, cytokine–cytokine receptor interaction, intestinal immune network for IgA production, glycine serine and threonine metabolism, and mismatch repair (Fig. 7B). Surprisingly, KEGG analysis showed that LACTB2 expression was correlated with many immune-related pathways, which can facilitate tumor progression. It may provide the potential mechanism for upregulated LACTB2 to cause poor prognosis. Additionally, coexpressing genes from string database were obtained and their interactions with LACTB2 are visualized in Fig. 7C.

### Correlation between LACTB2 expression and immune cell infiltration

**Fig. 8:**
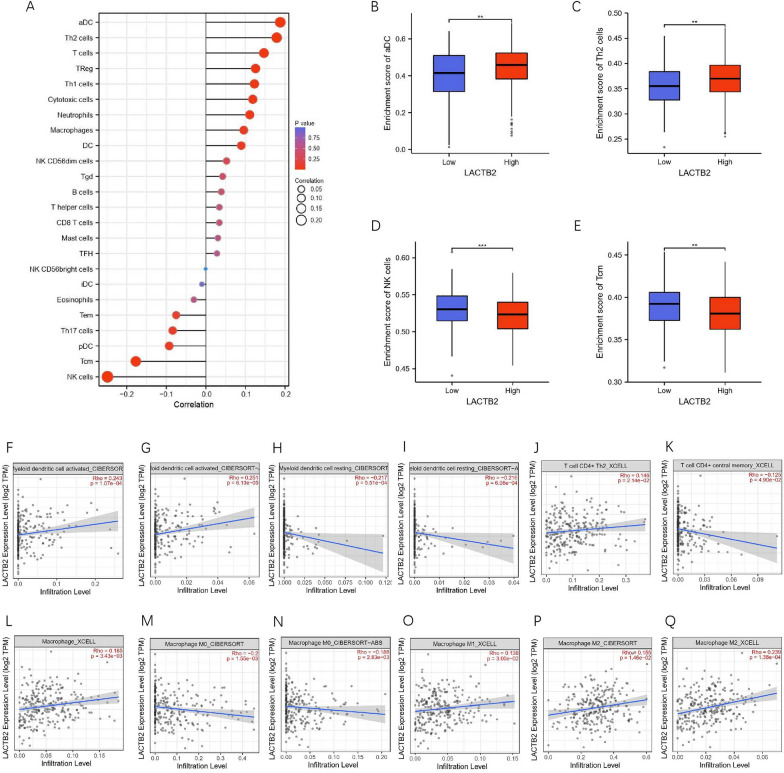
Immune infiltration analysis of LACTB2 in OC. **A** Lollipop chart of LACTB2 expression level in 24 types of immune cells. **B**–**E** Comparison of immune cells in low- and high-LACTB2 expression groups, including aDC (**B**), Th2 cells (**C**), NK cells (**D**), and Tcm (**E**). **F**–**I** There is a significant correlation between LACTB2 expression and dendritic cells, including the activated and the resting. **J**, **K** There is a significant correlation between LACTB2 expression and CD4+ T cells, including Th2 and central memory. **L**–**Q** There is a significant correlation between LACTB2 expression and macrophages, including M0, M1, and M2

Through the previous enrichment analysis, we found that LACTB2 was mainly associated with chemokine signaling pathway, primary immunodeficiency, cytokine–cytokine receptor interaction, and intestinal immune network for IgA production. These results indicated that LACTB2 may participate in immune processes in OC. Thus, we used the LACTB2 expression data from TCGA to analyze their relationship with 24 immune cells. LACTB2 expression showed significant correlation with aDC, Th2 cells, T cells, TReg, Th1 cells, cytotoxic cells, neutrophils, NK cells, and Tcm (Fig. 8A). Notably, aDC and Th2 cells had remarkably positive relationship with LACTB2 expression, while NK cells and Tcm showed negative relationship with LACTB2 expression. Further research demonstrated there were significant differences in immune cell infiltration levels, when LACTB2 expression was divided into high and low groups. LACTB2-high expression group had upregulated aCD and Th2 cells infiltration, and downregulated NK cells and Tcm infiltration (Fig. 8B–E). However, we found no significant correlation with NK cells using TIMER analysis (Additional file 1: Fig. S4E). Additionally, we analyzed the association of LACTB2 expression in OC with various immune cells using different algorithms (Fig. 8F–Q). The result revealed that the expression level of LACTB2 was negatively related with myeloid dendritic cell resting, T cell CD4+ central memory, and macrophage M0. In contrast, LACTB2 expression was positively related with myeloid dendritic cell activated, T cell CD4+ Th2, macrophage, macrophage M1, and M2. Finally, we investigated the relationship of LACTB2 expression and various T cells in pan-cancer level. The result showed that LACTB2 was positively associated with Th2 cells, but negatively associated with Th1 cells in most cancer types (Additional file 1: Fig. S4A). TIMER2 analysis was performed in SKCM, TGCT, and UVM patients to verify this relationship. The significantly negative correlation between LACTB2 expression and Th1 cells infiltration can be seen in all three cancers, while that of Th2 cells were positive (Additional file 1: Fig. S4B–D).

### Genetic alterations

We explore LACTB2 alteration, including gene mutation, copy number alterations, and structural variant, in pan-cancer level using cBioPortal. Gene alteration of LACTB2 was observed in various cancer types and the highest alteration frequency was in uterine carcinosarcoma. Alteration frequency of LACTB2 was relatively high in OC and the major type is amplification (Additional file 1: Fig. S2A). OncoPrint visual summary showed that 10% of OC cases had genetic alterations in LACTB2 (Additional file 1: Fig. S2C).

We then investigated the prognostic value of LACTB2 alteration in 33 cancers. We divided all cancer samples into LACTB2 alteration group and LACTB2 unalteration group. In altered group, DFS was significantly worse when comparing with unaltered group. That might indicate that LACTB2 alteration can lead to worse long-term outcomes of patients with malignant tumor (Additional file 1: Fig. S2B).

## Discussion

Ovarian carcinoma, as the most lethal gynaecological disease, causes more than 200,000 deaths among women annually [23]. Therefore, it is essential to establish a valid biomarker to facilitate early detection and intervention. LACTB2 participates in pathogenesis of many diseases, whereas its study in various tumors is relatively restricted. Hence, there is an urgent need to identify the impact of LACTB2 in the survival of cancer in terms of diagnosis, prognosis, progression and treatment. Previous studies clarified that LACTB2 is a mitochondrial endoribonuclease which can regulate mitochondrial function and promote tumor progression [5–7]. LACTB2 is demonstrated to be a prognostic indicator in colorectal cancer and nasopharyngeal cancer [8, 9]. Genetic alterations of LACTB2 expression were observed in colorectal cancer. Overexpression of LACTB2 can induce radioresistance in nasopharyngeal cancer patients. However, there is no study that has investigated the correlation between LACTB2 expression and OC. In this study, we have done a series of bioinformatics analyses to explore the prognostic value of LACTB2 and biological function in OC, especially its immune-related functions.

According to our results, pan-cancer analysis using TCGA data showed that LACTB2 expression was upregulated in 24 cancer types, including BLCA, BRCA, CESC, CHOL, COAD, DLBC, ESCA, GBM, HNSC, KIRP, LGG, LIHC, LUAD, LUSC, OC, PAAD, PRAD, READ, SKCM, STAD, THCA, THYM, UCEC, UCS. And it was significantly highly expressed in OC in the GSE12470, GSE18250, GSE137238, and GSE51088 datasets. In addition, ROC analysis reported an AUC = 0.981. These results may indicate that LACTB2 can be used as a screening marker to distinguish OC patients from healthy women. We further investigated the LACTB2 expression in OC based on various clinicopathological features. Patients with advanced OC have high expression of LACTB2. LACTB2 expression is higher in tumors with higher pathological grade. Similar results shown in protein expression analysis. In CTPAC database, the expression of LACTB2 was significantly increased in patients over 40 years. Furthermore, prognosis analysis demonstrated that higher LACTB2 expression was correlated with unfavorable prognosis in patients with tumors, such as OC, BRCA, and PAAD. OC patients with high LACTB2 expression have significantly worse OS, PFS, and RFS, compared with those who have low LACTB2 expression. These findings indicated that LACTB2 may be a promising candidate to predict the prognosis of OC patients.

Tumor Immune Microenvironment (TIME), as one of the important hallmarks of cancer, has been reported to be highly correlated with the development and progression of cancer [24]. Tumor-infiltrating immune cells are the significant components of TIME. Regulation of immune cells strongly influenced the TIME and the tumor behavior. Our study revealed the strong relationship between LACTB2 and TIME. GESA analysis showed that LACTB2 function was enriched in various immune-related processes, including chemokine signaling pathway, primary immunodeficiency, cytokine–cytokine receptor interaction, and intestinal immune network for IgA production. We next demonstrated that LACTB2 expression was strongly associated with the infiltration of multiple immune cells, including aDC, Th2, NK, Tcm. Thus, we hypothesized that LACTB2 may promote OC progression by regulating immune response, that is, the relative activation of immune cells. Recent study reported that LACTB2 participated in inflammatory response in Alzheimer’s disease [25]. As a mitochondrial protein, LACTB2 are supposed to have a role in mitochondrial mRNA turnover. According to previous research, mitochondria are important in immune cell regulation, which can influence immune cell metabolism, differentiation, activation of inflammatory responses and regulate transcription [26].

In pan-cancer analysis, we observed that LACTB2 expression has significantly positive relationship with Th2 cells, while have negative relationship with Th1 cells. Notably, our study also demonstrated that LACTB2 expression in OC was strongly positively related with Th2 cells infiltration. Th2 cells, as important components in TIME, have shown immunosuppressive effects, which in turn promote the development of cancer [27]. Hence, our study may indicate that LACTB2 can drive pro-tumoral immunosuppressive program in OC by regulating the tumor-infiltration of Th2 cells. Furthermore, a previous study demonstrated that increased level of Th2 cells induced recurrence in prostate cancer [28]. According to other studies, immune cell infiltration plays an important role in the overall survival of OC patients [10, 11]. In a recent study, researchers compared the Th2 cells infiltration between OC patients with poor response (PFS ≤ 6 months) and good response (PFS ≥ 12 months). The results showed that infiltration level of Th2 cells was significantly higher in group of poor response [29]. Considering the prognostic value of Th2 cells infiltration, LACTB2 may contribute to unfavorable prognosis in OC patients by inducing Th2 cell differentiation.

In conclusion, our study suggested that LACTB2 expression was upregulated in OC and indicated a poor prognosis. Additionally, we found that LACTB2 expression in OC has close relationship with various immune cells infiltration. Functional enrichment analysis also revealed that LACTB2 was associated with many immune-related processes. Consequently, LACTB2 may regulate the mechanism of tumor immunology to promote OC progression and lead to an unfavorable long-term outcome of patients. Our study reported the underlying pathways that LACTB2 may participate in and provide a promising immunotherapy target for OC. We hope that the results of our study will contribute to future research and assist clinicians to select proper interventions which can improve the prognosis of OC patients.

Although our study is innovative in investigating the association between LACTB2 expression and ovarian cancer, it has certain drawbacks. First, this study is mainly based on bioinformatics analysis, hence the majority of data were obtained from public databases. Further study can focus on in vivo or in vitro experiments, as well as clinical trials, to investigate the underlying mechanism of LACTB2 in OC. Second, a cohort of patients with long-term follow-up should be included in the prognostic analysis to validate the feasibility of LACTB2 as a prognostic biomarker. Third, subsequent studies should provide lager datasets to improve the reliability of the results. Certainly, our study has presented a new perspective on the role of LACTB2 in OC. However, more comprehensive and systematic researches are needed to validate our hypothesis in depth.

### Supplementary Information

**Additional file 1: Fig. S1.** ROC curve of LACTB2 in OC. X-axis represents false-positive rates, and Y-axis represents true-positive rates. **Fig. S2.** Genetic alteration analysis of LACTB2 in pan-cancer level. **(A)** Mutation types of LACTB2 in various cancers. **(B)** Correlation between LACTB2 alteration and DFS in pan-cancer analysis. **(C)** Summary of genetic alteration feature of LACTB2 in OC. **Fig. S3.** Correlation between LACTB2 expression and cancer patients’ survival. **(A, B)** Survival analysis of LACTB2 in BRCA. **(C, D)** Survival analysis of LACTB2 in HNSC. **(E, F)** Survival analysis of LACTB2 in PAAD. **(G)** Survival analysis of LACTB2 in ESCA. **(H)** Survival analysis of LACTB2 in UCEC. **Fig. S4.** Correlation between LACTB2 expression and immune cell infiltration in various cancers. **(A)** Heatmap of LACTB2 and different T cells CD4+ across 33 cancer types. **(B-D)** Purity-corrected Spearman’s correlation between two types of Th cells infiltration and LACTB2 expression in SKCM **(B)**, TGCT **(C)**, and UVM **(D)**. **(E)** TIMER2 analysis of the relationship between LACTB2 and NK cells in OC. **Table S1.** The full names of tumor abbreviation from TCGA.

## Data Availability

The data and materials of this study were obtained from publicly accessible databases. The data presented in this study are openly available in TCGA dataset (https://www.ncbi.nlm.nih.gov/geo/) and GEO dataset (https://portal.gdc.cancer.gov/).
