# Supplementary material for: Identification of the prognostic value of LACTB2 and its correlation with immune infiltrates in ovarian cancer by integrated bioinformatics analyses
Source: Eur J Med Res. 2024 Mar 12;29:166. doi: 10.1186/s40001-024-01762-2 (PMC10929091; doi:10.1186/s40001-024-01762-2)
Supplement: Supplementary file 1 — Additional file 1: Fig. S1. ROC curve of LACTB2 in OC. X-axis represents false-positive rates, and Y-axis represents true-positive rates. Fig. S2. Genetic alteration analysis of LACTB2 in pan-cancer level. (A) Mutation types of LACTB2 in various cancers. (B) Correlation between LACTB2 alteration and DFS in pan-cancer analysis. (C) Summary of genetic alteration feature of LACTB2 in OC. Fig. S3. Correlation between LACTB2 expression and cancer patients’ survival. (A, B) Survival analysis of LACTB2 in BRCA. (C, D) Survival analysis of LACTB2 in HNSC. (E, F) Survival analysis of LACTB2 in PAAD. (G) Survival analysis of LACTB2 in ESCA. (H) Survival analysis of LACTB2 in UCEC. Fig. S4. Correlation between LACTB2 expression and immune cell infiltration in various cancers. (A) Heatmap of LACTB2 and different T cells CD4+ across 33 cancer types. (B-D) Purity-corrected Spearman’s correlation between two types of Th cells infiltration and LACTB2 expression in SKCM (B), TGCT (C), and UVM (D). (E) TIMER2 analysis of the relationship between LACTB2 and NK cells in OC. Table S1. The full names of tumor abbreviation from TCGA. [file 40001_2024_1762_MOESM1_ESM.docx]

**Additional file**

**
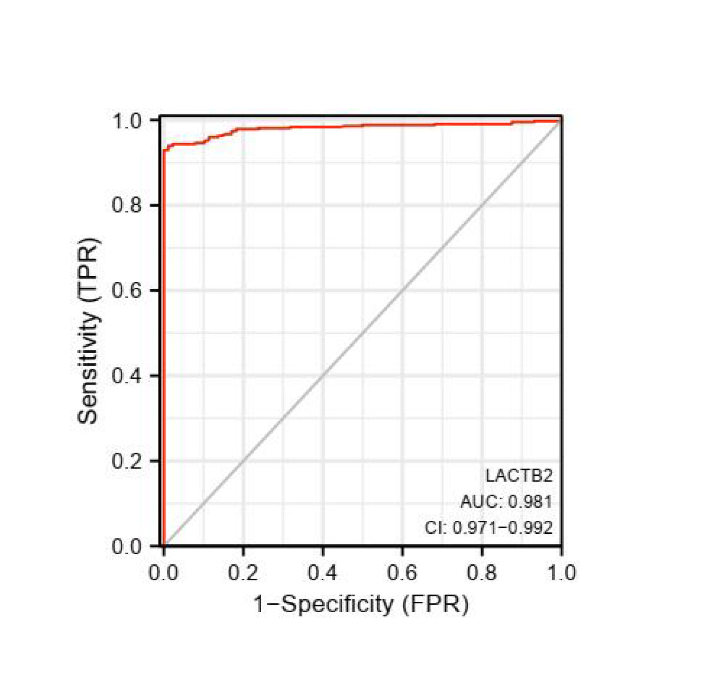
**

**Fig. S1** ROC curve of LACTB2 in OC. X-axis represents false-positive rates, and Y-axis represents true-positive rates.


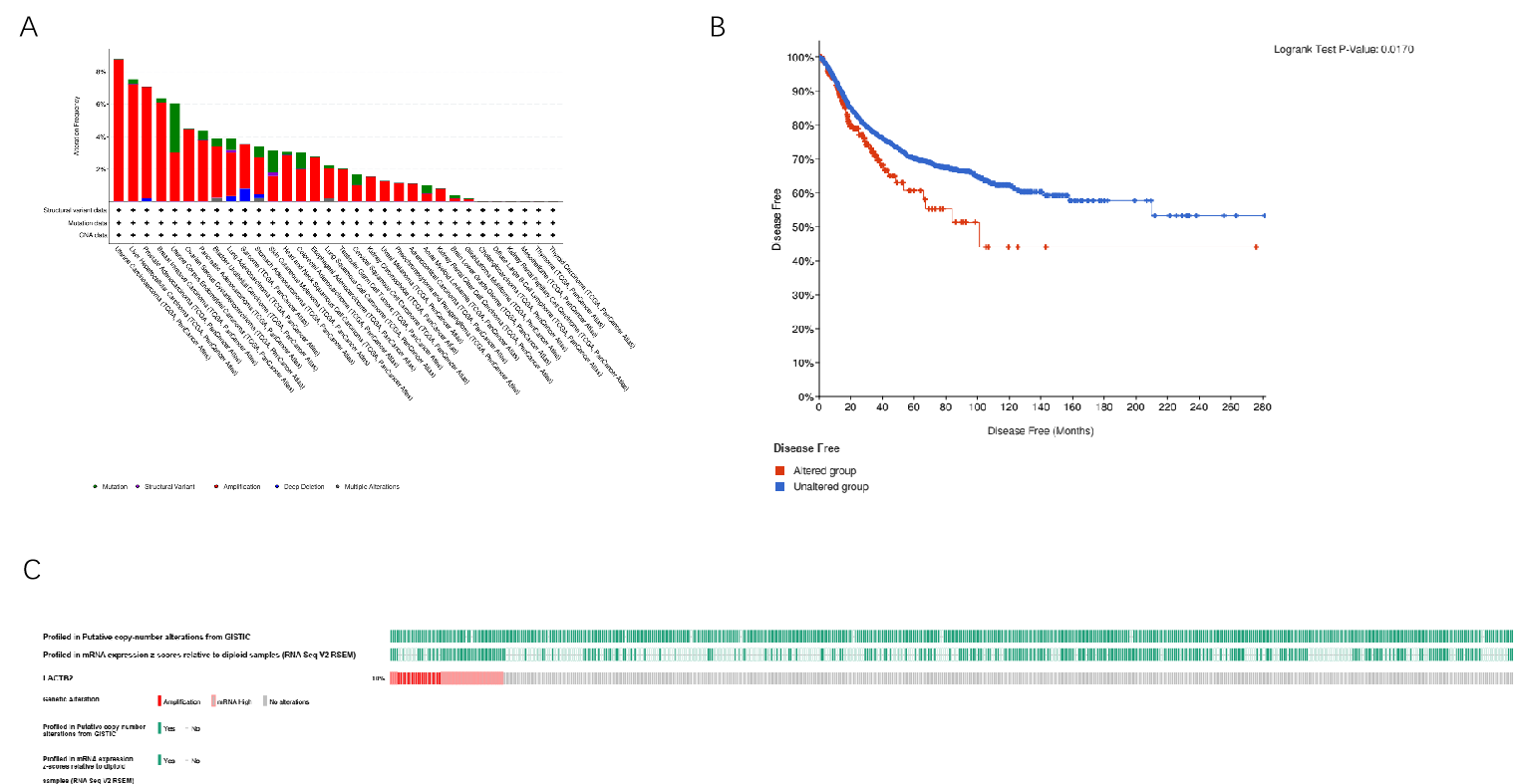


**Fig. S2** Genetic alteration analysis of LACTB2 in pan-cancer level. **(A)** Mutation types of LACTB2 in various cancers. **(B)** Correlation between LACTB2 alteration and DFS in pan-cancer analysis. **(C)** Summary of genetic alteration feature of LACTB2 in OC.

**
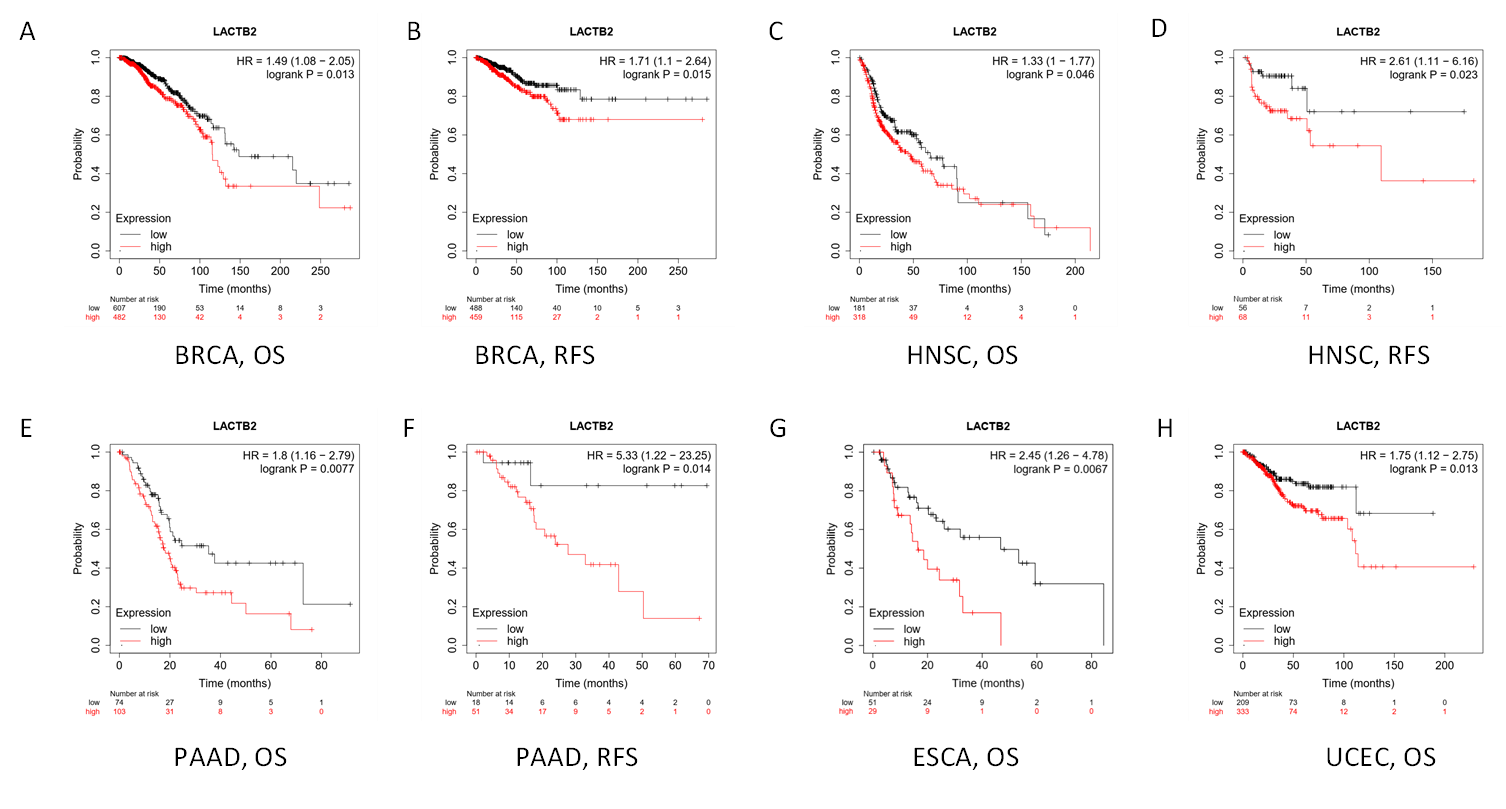
**

**Fig. S3** Correlation between LACTB2 expression and cancer patients’ survival. **(A, B)** Survival analysis of LACTB2 in BRCA. **(C, D)** Survival analysis of LACTB2 in HNSC. **(E, F)** Survival analysis of LACTB2 in PAAD. **(G)** Survival analysis of LACTB2 in ESCA. **(H)** Survival analysis of LACTB2 in UCEC.

**
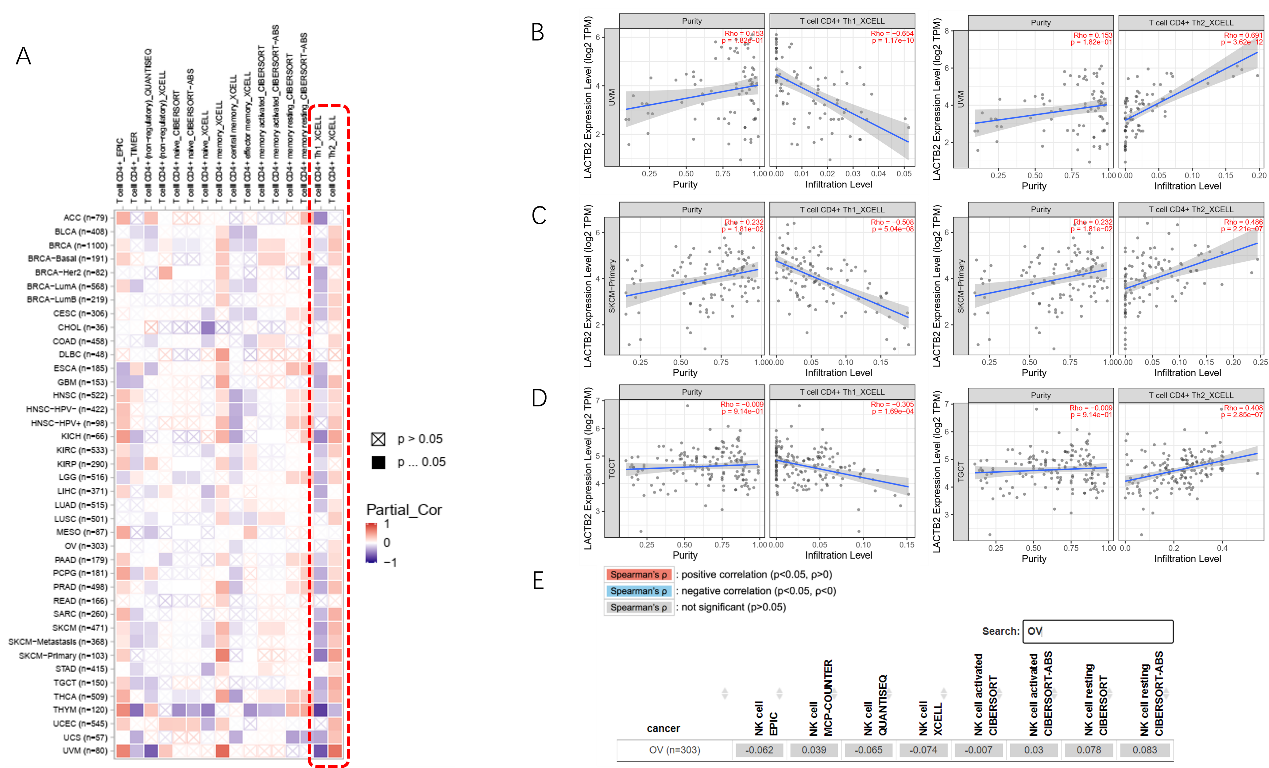
**

**Fig. S4** Correlation between LACTB2 expression and immune cell infiltration in various cancers. **(A)** Heatmap of LACTB2 and different T cells CD4+ across 33 cancer types. **(B-D)** Purity-corrected Spearman’s correlation between two types of Th cells infiltration and LACTB2 expression in SKCM **(B)**, TGCT **(C)**, and UVM **(D)**. **(E)** TIMER2 analysis of the relationship between LACTB2 and NK cells in OC.

**Table S1. The full names of tumor abbreviation from TCGA.**

| Abbreviations | Full name |
| --- | --- |
| ACC | Adrenocortical carcinoma |
| BLCA | Bladder Urothelial Carcinoma |
| BRCA | Breast invasive carcinoma |
| CESC | Cervical squamous cell carcinoma and endocervical adenocarcinoma |
| CHOL | Cholangiocarcinoma |
| COAD | Colon adenocarcinoma |
| DLBC | Lymphoid Neoplasm Diffuse Large B-cell Lymphoma |
| ESCA | Esophageal carcinoma |
| GBM | Glioblastoma multiforme |
| HNSC | Head and Neck squamous cell carcinoma |
| KICH | Kidney Chromophobe |
| KIRC | Kidney renal clear cell carcinoma |
| KIRP | Kidney renal papillary cell carcinoma |
| LAMI | Acute Myeloid Leukemia |
| LGG | Lower Grade Glioma |
| LIHC | Liver hepatocellular carcinoma |
| LUAD | Lung adenocarcinoma |
| LUSC | Lung squamous cell carcinoma |
| MESO | Mesothelioma |
| OV | Ovarian serous cystadenocarcinoma |
| PAAD | Pancreatic ductal adenocarcinoma |
| PCPG | Pheochromocytoma and Paraganglioma |
| PRAD | Prostate adenocarcinoma |
| READ | Rectum adenocarcinoma |
| SARC | Sarcoma |
| SKCM | Skin Cutaneous Melanoma |
| STAD | Stomach adenocarcinoma |
| TGCT | Testicular Germ Cell Tumor |
| THCA | Thyroid carcinoma |
| THM | Thymoma |
| UCEC | Uterine Corpus Endometrial Carcinoma |
| UCS | Uterine Carcinosarcoma |
| UVM | Uveal Melanoma |
